# Effect of Chitosan as a Cross-Linker on Matrix Metalloproteinase Activity and Bond Stability with Different Adhesive Systems

**DOI:** 10.3390/md18050263

**Published:** 2020-05-18

**Authors:** Eugenia Baena, Sandra R Cunha, Tatjana Maravić, Allegra Comba, Federica Paganelli, Giulio Alessandri-Bonetti, Laura Ceballos, Franklin R Tay, Lorenzo Breschi, Annalisa Mazzoni

**Affiliations:** 1 Area of Stomatology, Health Sciences Faculty, King Juan Carlos University, Avda. de Atenas, s/n. 28922 Alcorcón, Madrid, Spain; eugenia.baena@urjc.es (E.B.); laura.ceballos@urjc.es (L.C.); 2 Department of Biomedical and Neuromotor Sciences, DIBINEM, University of Bologna, Alma Mater Studiorum, Via San Vitale 59, 40125 Bologna, Italy; sandra-cunha@uiowa.edu (S.R.C.); tatjana.maravic2@unibo.it (T.M.); allegra.comba@unibo.it (A.C.); paganellifederica73@gmail.com (F.P.); giulio.alessandri@unibo.it (G.A.-B.); annalisa.mazzoni@unibo.it (A.M.); 3 Department of Restorative Dentistry, School of Dentistry, University of São Paulo, São Paulo 14040-904, Brazil; 4 Department of Operative Dentistry, College of Dentistry, University of Iowa, 801 Newton Rd, Iowa City, IA 52242, USA; 5 School of Dentistry, Faculty of Medicine, University of Novi Sad, Hajduk Veljkova 3, 21000 Novi Sad, Serbia; 6 Department of Endodontics, The Dental College of Georgia, Augusta University, 1430 John Wesley Gilbert Drive, Augusta, GA 30912, USA; ftay@augusta.edu

**Keywords:** chitosan, matrix metalloproteinases, bond strength, cross-linkers, zymography

## Abstract

The aim of the present study was to evaluate the effect of 0.1% chitosan (Ch) solution as an additional primer on the mechanical durability and enzymatic activity on dentine using an etch-and-rinse (E&R) adhesive and a universal self-etch (SE) adhesive. Microtensile bond strength and interfacial nanoleakage expression of the bonded interfaces for all adhesives (with or without pretreatment with 0.1% Ch solution for 1 min and air-dried for 5 s) were analyzed immediately and after 10,000 thermocycles. Zymograms of protein extracts from human dentine powder incubated with Optibond FL and Scotchbond Universal on untreated or Ch-treated dentine were obtained to examine dentine matrix metalloproteinase (MMP) activities. The use of 0.1% Ch solution as an additional primer in conjunction with the E&R or SE adhesive did not appear to have influenced the immediate bond strength (T0) or bond strength after thermocycling (T1). Zymography showed a reduction in MMP activities only for mineralized and demineralized dentine powder after the application of Ch. Application of 0.1% Ch solution does not increase the longevity of resin–dentine bonds. Nonetheless, the procedure appears to be proficient in reducing dentine MMP activities within groups without adhesive treatments. Further studies are required to comprehend the cross-linking of Ch with dentine collagen.

## 1. Introduction

Adhesion to dentine relies on the formation of the hybrid layer (HL), a structure consisting of an adhesive resin monomer-infiltrated demineralized dentine collagen matrix [1,2,3]. This connecting layer is the weakest link in the resin–dentine interface because collagen fibrils that are not completely infiltrated by resin are susceptible to enzymatic degradation over time [4,5,6]. 

The most prominent group of endogenous enzymes in dentine are the matrix metalloproteinases (MMPs). These enzymes are a family of Zn^2+^- and Ca^2+^-dependent proteases that are present in mineralized dentine and the dentinal tubules [7,8,9,10]. Endogenous proteases are trapped by apatite crystallites in the dentine collagen matrix in non-functional forms. When exposed to a low pH environment, such as those created by etch-and-rinse or self-etch adhesive systems, the MMPs are exposed and converted to active functional forms [11,12,13,14]. Inhibition of collagen degradation by MMPs improves HL integrity, decreases nanoleakage, and enhances the durability of resin–dentine bonds [1,15]. 

Several mechanisms have been proposed to reduce HL degradation over time. The use of cross-linking agents prior to adhesive application improves collagen stiffness through the foundation of additional hydrogen bonding and/or formation of covalent intermolecular and intramolecular cross-links [1,4,16,17,18,19,20,21]. Although an extensive range of cross-linking agents are available, the specific anti-MMP effects of these agents remain obscure.

Recently, research on novel materials in the biomedical field [22,23,24] has shifted towards nature-derived materials (such as marine-based materials) obtained using advanced eco-friendly technologies [25]. One of the materials of interest in the field of dentistry is chitosan (Ch), a natural hydrophilic polycationic biopolymer derived from alkaline deacetylation of chitin. It is present in the shells of crustaceans and has a broad range of potential dental applications because of its biocompatibility, adhesive potential, and antibacterial properties [26,27,28,29]. Ch has been described in the literature as a biopolymer with cross-linking properties because of its large number of free hydroxyl and amino groups that form ionic complexes with collagen. Cross-linking dentine collagen with Ch produces a micro-fibrillar network with superior mechanical properties. These cross-linked collagen matrices also possess antimicrobial and anti-biofilm activities [28,30,31,32]. Incorporation of Ch into dentine adhesives apparently did not interfere with the microtensile bond strength of those adhesives to dentine; such a regime also reduced the adverse effects of ageing of the resin–dentine interface by thermo-mechanical cycling and created interfaces with antibacterial properties [33,34]. When Ch was used with riboflavin, an MMPs inhibitor, the combination improved the mechanical properties of dentine and reduced the degradation of the resin–dentine interface synergistically [28,35].

Accordingly, the objective of the present study was to examine the effect of using 0.1% Ch solution as an additional primer on the bond strength of resin-bonded dentine created with a three-step etch-and-rinse adhesive or a universal self-etch adhesive. Zymography of dentine extracts was also performed to analyze the potential inhibition effect of Ch on dentine MMPs. The null hypotheses tested were that the use of Ch as a collagen cross-linker: (1) Has no effect on preserving the resin–dentine bond strength over time, and (2) has no effect on the inactivation of endogenous dentine MMPs.

## 2. Results

### 2.1. Microtensile Bond Strength Test (µTBS) 

The sample preparation procedures for the different groups are reported in Table 1. The microtensile bond strengths of the four experimental groups at T0 and T1 are shown in Table 2. Both the factors adhesive (*p* < 0.001) and time (*p* < 0.001) significantly affected the bond strength results. The interaction of these two factors was also significant (*p* < 0.001). At T0, Optibond FL (OFL) and Optibond FL with chitosan pretreatment (OFL-Ch) showed the highest µTBS values, with no statistical differences between the two experimental subgroups. Scotchbond Universal (SBU) µTBS was not significantly different from the µTBS of Scotchbond Universal with chitosan pretreatment (SBU-Ch). According to these results, the use of 0.1% Ch as an additional primer did not adversely affect the immediate bond strength of both adhesives immediately after bonding. At T1, all adhesives presented similar µTBS values regardless of Ch pre-treatment, with no statistical differences among the four T1 experimental subgroups. After thermocycling, the bond strength of OFL and OFL-Ch decreased, differently from the SBU and SBU-Ch groups that had their µTBS values increased. 

### 2.2. Interfacial Nanoleakage 

**Figure 1:**
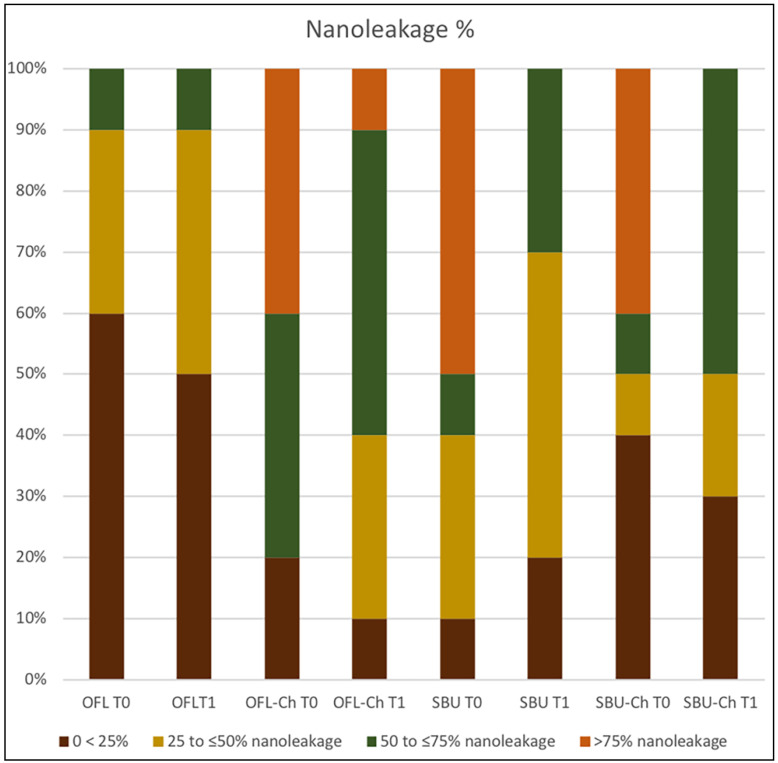
Distribution of the nanoleakage expression (%) for each experimental group at times T0 and T1. OFL: Optibond FL; SBU: Scotchbond Universal; Ch.

The four 2 × 4 contingency tables depicting the nanoleakage scores from the four subgroups are shown in Table 3, Table 4, Table 5 and Table 6. For OFL and OFL-Ch, the sum of the probabilities of the observed array of cell frequencies, together with the probabilities of all other cell frequency arrays that are less than or equal to the probability of the observed array (i.e., PA) for T0 is 0.0095 and for T1 0.0069. There was a statistically significant association between the method in which the specimens were bonded and the nanoleakage score (*p* < 0.05). Nevertheless, for SBF and SBF-Ch, the probability of the observed array of cell frequencies plus the sum of the probabilities of all other cell frequency arrays are equal to or smaller than the probability of the observed array (i.e., PA) for T0 is 0.5726 and for T1 05226. A statistically significant association between the method in which the specimens were bonded and the nanoleakage score was not found (*p* > 0.05). The percentage distribution of nanoleakage is shown in Figure 1.

**Figure 2:**
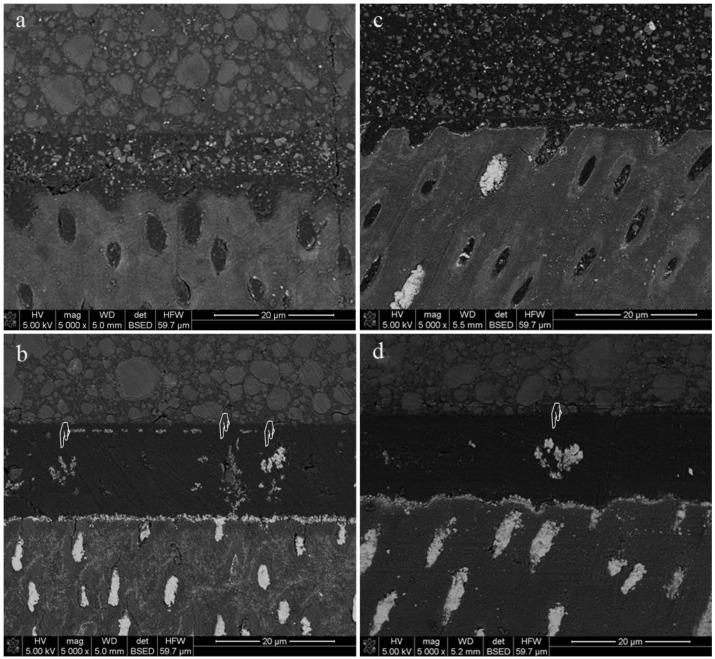
Scanning electron microscopy images of representative nanoleakage expression (pointers) along the resin–dentine interface created by different adhesives in the presence or absence of Ch pretreatment. OFL: Optibond FL; SBU: Scotchbond Universal. (**a**) OFL at time T0. (**b**) OFL-Ch at time T0. A spotted pattern was identified for these experimental subgroups. (**c**) SBU at time T0. (**d**) SBU-Ch at time T0. A more defined linear nanoleakage pattern was observed for these subgroups. Silver depositions were more continuous and water channels (i.e., water channels; pointers) were detected within the adhesive layers.

Representative scanning electron microscopy images of the nanoleakage expression along the resin–dentine interfaces of the four experimental groups at time T0 are shown in Figure 2. The OFL (Figure 2a) and OFL-Ch (Figure 2b) groups showed dispersed silver deposition with a spotted pattern. Conversely, specimens bonded with the universal adhesive SBU exhibited more continuous and linear silver deposition along the interface together with a reticular pattern within the resin. Silver-impregnated water channels (i.e., water trees) were evident in specimens bonded with SBU regardless of Ch application (Figure 2c,d). 

### 2.3. Zymography

**Figure 3:**
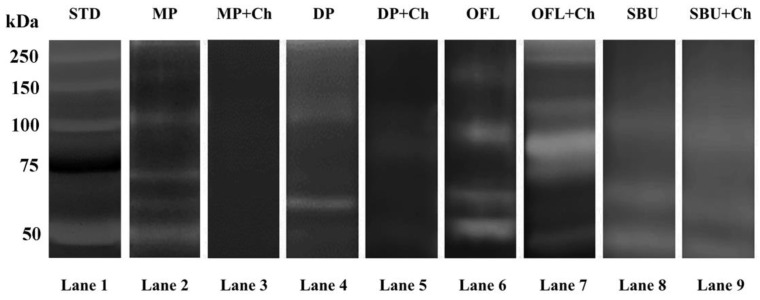
Zymography of extracts obtained from dentine powder treated with the two adhesives with or without Ch pre-treatment. Lane 1: Standards (Std) in kDa. Lane 2: mineralized dentine powder (MP). Lane 3: mineralized dentine powder after incubation with Ch. Lane 4: dentine powder demineralized with 10% phosphoric acid (DP). Lane 5: demineralized dentine powder treated with Ch. Lane 6: demineralized dentine powder treated with OFL. Lane 7: demineralized dentine powder incubated with Ch followed by OFL adhesive application. Lane 8: mineralized dentine powder incubated with Ch followed by SBU adhesive application. Lane 9: mineralized dentine powder incubated with Ch followed by SBU adhesive application.

Bands around 72, 67, and 92 kDa, corresponding to the molecular weight of the pro- and active form of MMP-2 and pro-MMP-9, respectively, were present in the positive control (MP), along with an additional band around 45 kDa that might represent the degradation products of MMP-2 or MMP-9 (Figure 3, lane 2) [36]. Incubation of mineralized dentine with 0.1% Ch (MP + Ch) inactivated the enzymatic activities completely (Figure 3, lane 3).

Gelatinolytic activities were also detected in the demineralized dentine powder, with increases in MMP-2 and pro-MMP-9 expression, when compared to the mineralized dentine powder (Figure 3, lane 4). After the incubation of demineralized dentine powder with 0.1% Ch, the expression of pro-MMP-2 and pro-MMP-9 decreased and MMP-2 was no longer detectable (Figure 3, lane 5). 

Application of the etch-and-rinse adhesive OFL to 10% phosphoric acid-etched dentine powder (Figure 3, lane 6) and pretreatment of the acid-etched dentine powder with Ch prior to OFL application (Figure 3, lane 7) activated MMP-2 and MMP-9, with the appearance of the active form of MMP-9 at 86 kDa [36]. When mineralized dentine powder was treated with the universal adhesive SBU in self-etch mode, pro-MMP-9, MMP-2, and a band around 50 kDa were detected in the zymogram (Figure 3, lane 8). Pretreatment of the mineralized dentine powder with 0.1% Ch prior to the application of SBU in the self-etch mode did not influence the enzymatic activity (Figure 3, lane 9). 

## 3. Discussion

The present study used a 0.1% Ch solution as an additional primer before dentine bonding procedures. Application of Ch before bonding with an etch-and-rinse adhesive or a self-etch adhesive to dentine did not result in better preservation of the μTBS immediately as well as after thermocycling. Thus, the first null hypothesis that “the use of Ch as a collagen cross-linker has no effect on bond strength deterioration over time” cannot be rejected. Based on the results derived from zymography of the dentine powder extracts, the use of 0.1% Ch solution decreased the enzymatic activity of MMPs in some groups. Thus, the second null hypothesis that “Ch as a collagen cross-linker has no effect on inactivation of endogenous dentine MMPs” has to be partially rejected.

The use of aldehydes, such as glutaraldehyde or acrolein, as cross-linking agents for demineralized dentine collagen matrices has been reported previously [20,37,38,39]. The aldehyde group increases the strength of collagen by chemically interacting with its amino groups, thereby minimizing the degradation of the adhesive interface. Despite the effectiveness of aldehydes in preserving the HL, they cannot be used clinically because of their extreme cytotoxicity caused by depolymerization [1,40,41]. 

The use of natural less toxic polymers for dentine pre-treatment, such as Ch, has recently been studied [4,33,34,42]. Previous reports stated that the incorporation [34] of this polymer within an adhesive did not influence the immediate bond strength. Conversely, the addition of Ch conferred additional benefits, such as antibacterial activity or delayed thermo-mechanical ageing [33,34]. Similarly, results from the present study indicate that the immediate and thermocycled bond strengths of the adhesives tested were not adversely affected by the use of Ch for dentine pre-treatment. It is interesting that the effect of thermocycling is adhesive dependent; the tbond strengths of the OFL and OFL-Ch experimental groups decreased while those of the SBU and SBU-Ch groups increased at T1.

The difference between the bond strengths of the etch-and-rinse and self-etch adhesives has already been discussed in the literature. Potential clinical errors may arise upon the use of phosphoric acid-etching because of the need to rinse off the etchant and the difficulty in managing dentine moisture during the application of etch-and-rinse adhesives. Self-etch adhesives containing 10-methacryloyloxydecyl dihydrogen phosphate, such as SBU, anecdotally provides a more stable chemical bond to dentine over the course of time [43,44,45]. Deposition of stable 10-MDP-Ca salt in Hydroxyethylmethacrylate (HEMA)-free adhesives [46], via the formation of a nanolayer at the adhesive interface formed by this monomer, conceptually increases the interfacial mechanical strength [45,47,48]. Unfortunately, nanolayering of 10-MDP-Ca salt is rarely identified in resin–dentine interfaces created by contemporary 10-MDP-containing adhesives [49] and the incorporation of 10-MDP in adhesives does not contribute to the durability of resin–dentine bonds [50]. Conversely, interfacial stress relaxation via hygroscopic expansion of the overlying composite may produce an increase in the bond strength over a certain period of time [51]. Self-etch adhesives, which are acidic in nature, require a relatively longer time to achieve adequate degrees of conversion [51,52]. Such a phenomenon provides rational justification of the higher bond strengths values observed in the SBU groups after thermocycling.

Nanoleakage examination identified nanometre-sized defects within the resin–dentine interface that increased with ageing [53,54]. These defects represent water-rich regions that resin has failed to infiltrate, and are the locations where degradation of the HL occurs over time [50]. In the present study, the three-step etch-and-rinse adhesive OFL had lower nanoleakage scores compared with the SBU universal adhesive. Three-step etch-and-rinse adhesives contain a lower percentage of hydrophilic resin monomers compared to two-step etch-and-rinse and one-step self-etch adhesives. This implies that three-step adhesive systems produce less permeable resin–dentine interfaces after in situ polymerization, and, hence, lower nanoleakage expression [55,56,57]. When Ch solution was used as an additional primer with OFL, it may have increased the hydrophilic properties of the adhesive system, providing a more porous HL. Regardless of the bonding system employed and the number of steps required for its application, all adhesive systems examined exhibited variable degrees of incomplete polymerization [57,58,59]. 

The results of the present study confirm that MMP activation is induced by both etch-and-rinse and universal adhesives, although higher levels of activity were detected for the etch-and-rinse adhesive. The etching step of the etch-and-rinse adhesives exposes a more apatite-depleted collagen dentine matrix than self-etch adhesives [1]. In addition, collagen matrices exposed by self-etched adhesives are partially protected by intrafibrillar apatite crystallites. Hence, more extensive MMP activation is expected with the use of etch-and-rinse adhesives [60]. The activity induced by OFL and SBU was not completely inhibited after Ch application, despite the observation that Ch was able to inactivate MMP activity on mineralized and demineralized dentine powder. 

Apart from increasing the collagen stiffness through the creation of interfibrillar and intrafibrillar cross-links, the inactivation of endogenous MMPs may also be a property of the cross-linking agents [12,20,21,61,62]. Cross-linking agents inactivate dentine MMPs exposed by acid demineralization by altering the 3-D conformation of the catalytic domains of those enzymes, preventing them from actively interacting with the collagen substrate [54]. Despite previous studies demonstrating that the use of Ch with carbodiimide, N-hydroxysuccinimide, and riboflavin improves the resistance of dentine surface collagen to enzymatic degradation, this effect could not be identified [30,63,64]. The present study is the first one to use Ch solution as a dentine primer without incorporating the polymer with other cross-linking agents. The inability of Ch solution to reduce endogenous protease activity in the adhesive groups may be attributed to (1) undesirable interaction between the adhesive systems with Ch that may have compromised the function of Ch as a cross-linking agent; and (2) Ch, when used alone, is a weak cross-linker, because it only inhibits MMPs when applied on mineralized and demineralized dentine without adhesive systems. The initial cross-linking effect of Ch is probably futile when the pro-forms of the MMPs are activated to their active forms by acidic adhesive resin monomers. Further in vivo studies should clarify the role Ch in stabilizing the adhesive interface.

## 4. Materials and Methods 

### 4.1. Microtensile Bond Strength (µTBS) 

Sixty recently extracted human molars were sectioned with a slow-speed diamond blade (Isomet 5000, Buehler Ltd., Lake Buff, IL, USA), under water cooling, into 4-mm-thick dentine discs comprising deep coronal dentine. A standardized smear layer was created with 600-grit silicon carbide paper (Buehler Ltd.). The specimens were divided into 4 experimental groups (*n* = 10) according to the type of adhesive system and whether 0.1% Ch was employed for dentine pretreatment: Group 1: OFL (*n* = 10): Dentine was etched for 15 seconds with 37.5% phosphoric-acid gel (Kerr, Orange, CA, USA) and rinsed with water. Acid-etched dentine was primed and bonded with Optibond FL (Kerr) following the manufacturer’s instructions; Group 2: OFL-Ch (*n* = 10): Dentine was etched as for group 1. Acid-etched dentine was pretreated with 0.1% Ch water-solution for 1 min and air-dried for 5 s. The Ch-treated dentine was subsequently primed and bonded with Optibond FL; Group 3: SBU (*n* = 10): Scotchbond Universal (3M ESPE, St. Paul, MN, USA) was applied in self-etch mode on mineralized dentine according to the manufacturer’s instructions; Group 4: SBU-Ch (*n* = 10): Mineralized dentine was treated with 0.1 % Ch for 1 min and air-dried for 5 seconds. Scotchbond Universal adhesive was then applied as for group 3. 

Two-millimeter-thick layers of a nanofilled resin composite (Filtek Supreme XTE, 3M ESPE, St. Paul, MN, USA) were placed and light-cured for 40 seconds (Elipar, 3M ESPE, St. Paul, MN, USA). The teeth were either stored in artificial saliva for 24 h [11] (*n* = 5; T0) or thermocycled (*n* = 5; T1; 10,000 cycles; 5 °C/55 °C, 30 s of exposure time) before microtensile testing. Resin-dentine sticks were made with cross-sectional areas of 0.8 mm × 0.8 mm from each bonded tooth, using a low-speed saw with water cooling. Each stick had its dimension measured with a pair of digital calipers (±0.01 mm) and was stressed to failure using a simplified universal testing (Instron 3345, Instron Corp., Canton, MA, USA) at a crosshead speed of 1 mm/min. Failure mode was evaluated using a stereomicroscope (SZX7, Olympus Inc., Hamburg, Germany) at 30× magnification and classified as adhesive (A), cohesive in composite (CC), cohesive in dentine (CD), or mixed (M) failures. Statistical analysis was performed by two-way analysis of variance to examine the effects of adhesive treatment and thermocycling, and the interaction of these two factors on bond strength. Post-hoc pairwise comparisons were performed using the Tukey test. For all analyses, statistical significance was set at *α* = 0.05.

### 4.2. Interfacial Nanoleakage 

At baseline (T0) or after thermocycling (T1), 2 sticks from each tooth prepared for the microtensile testing were selected for nanoleakage evaluation (*n* = 5). Specimens were covered with nail varnish, leaving 1 mm of dentine or resin composite exposed on either side of the bonded interface. The varnished-covered specimens were immersed in 50 wt% ammoniacal silver nitrate for 24 h at 37 °C in a light-proof container [65]. After 24 h, the specimens were rinsed with distilled water for 1 min and immersed in a photodeveloping solution for 8 hours under a fluorescent light to reduce silver ions into metallic silver grain within voids along the bonded interfaces. The sticks were polished under water, with 600 grit SiC discs, to remove the varnish coats and were embedded in transparent chemically cure epoxy resin (Epoxycure, Buehler Ltd., Lake Bluff, IL, USA). The embedded specimens were reduced to half of its thickness with SiC discs and the exposed surface was mechanically polished with a wet # 600, 800, 1200, and 4000 grit SiC discs (Buehler Ltd., Lake Bluff, IL, USA). Thereafter, the specimens were ultrasonically cleaned for 5 minutes, air dried, and mounted on stubs. Resin–dentine interfaces were analyzed by a field emission scanning electron microscope operated in the backscattered mode (Phillips XL30 ESEM, FEI Company, Hillsboro, OR, USA). The extent of interfacial nanoleakage along the resin interface was scored on a 4-point scale by two observers: 0%–25% nanoleakage, 25%–50% nanoleakage, 50%–75% nanoleakage, and 75%–100% nanoleakage. Representative specimens were coated with carbon (Bal-Tec Sputter Coater SCD 005, Bal-Tec GmbH, Witten, Germany) and examined using a scanning electron microscope (UHR Nova NanoSEM 230, FEI Company, Hillsboro, OR, USA). 

Nanoleakage scores in the 4 subgroups were arranged into four 2 × 4 contingency table and analyzed with the Fisher–Freeman–Halton statistic [66]. 

### 4.3. Zymography of Dentine Extracts 

Zymographic was performed according to Mazzoni et al. [60]. Mineralized dentine powder was obtained from an additional 12 humans’ third molars after freezing the dentine in liquid nitrogen and triturating the frozen mineralized dentine chips using a Retsch mill (Model MM400, Retsch GmbH, Haan, Germany). Aliquots of mineralized dentine powder were divided into 8 groups (Table 1):

- Group 1 (Lane 1―MP): Untreated mineralized dentine powder (MP; control).
- Group 2 (Lane 2―MP + Ch): MP was treated with 0.1% Ch for 30 min.
- Group 3 (Lane 3―DP): MP treated with 1 mL of 10 wt% phosphoric acid for 10 min and used as demineralized control (DP).
- Group 4 (Lane 4―DP + Ch): DP was treated with 0.1% Ch for 30 min.
- Group 5 (Lane 5―OFL): DP was mixed with 100 µL of Optibond FL (OFL).
- Group 6 (Lane 6―OFL + Ch): DP was treated with 0.1% Ch and then mixed with 100 µL of OFL.
- Group 7 (Lane 7―SBU): MP was mixed with 100 µL of Scotchbond Universal (SBU).
- Group 8 (Lane 8―SBU + Ch): MP was mixed with 0.1% Ch and then mixed with 100 µL of SBU.

For the adhesive groups, 1 mL of acetone was used to extract the adhesive from the treated dentine powder. The extract was subsequently centrifuged (20,800× *g* for 20 min), re-suspended in acetone, and re-centrifuged 2 more times for the removal of additional unpolymerized comonomers. 

Dentine powder aliquots were re-suspended in extraction buffer (50 mM Tris–HCl, pH 6, containing 5 mM CaCl_2_, 100 mM NaCl, 0.1% Triton X-100, 0.1% non-ionic detergent P-40, 0.1 mM ZnCl_2_, 0.02% NaN_3_) for 24 h at 4 °C. The mixture was sonicated intermittently for 10 minutes (*ca.* ≈30 pulses), and centrifuged for 20 min at 4 °C (12,000 rpm). The supernatant was removed and re-centrifuged. The protein content was concentrated using a centrifugal concentrator (Vivaspin 500, 10,000kDa cut off; Sartorius Stedim Biotech, Goettingen, Germany) for 30 min at 4 °C (10,000 rpm for 3 times). Total protein concentration was determined using the Bradford assay (Bio-Rad, Hercules, CA, USA) with a spectrophotometer. Dentine protein aliquots (60 μg) were diluted in Laemmli sample buffer in a 4:1 ratio. Sodium dodecyl sulfate-polyacrylamide gel electrophoresis (SDS-PAGE) was then performed in plates containing 1 mg/mL fluorescein-labelled gelatine. Pre-stained low-range molecular-weight SDS-PAGE standards (Bio-Rad) were used as molecular-weight markers. After electrophoresis, the gels were rinsed for 1 h in 2% Triton X-100 and incubated in zymography activation buffer (50 mmol/L Tris–HCl, 5mmol/L CaCl_2_, pH 7.4) for 48 h. Proteolytic activities were recorded using a long-wave ultraviolet light scanner (ChemiDoc Universal Hood, Bio-Rad).

## 5. Conclusions

Within the limitations of the present study (i.e., in vitro), it may be concluded that the use of 0.1% Ch solution as an additional primer does not influence the durability or bond strength of the resin–dentine bond over time, as applied to the etch-and-rinse and self-etch adhesives examined. Dentine collagen cross-linking produced by Ch appears to be efficient only when adhesives are not used. Further studies are required to fully understand the cross-linking of Ch with dentine and its possible interaction with adhesive systems.

## Figures and Tables

**Table 1:** Groups and clinical procedure for microtensile bond strength test.

	Sample Preparation
**G1: OFL**	Dentine etching for 15 s with 37.5% phosphoric-acid gel (Kerr, Orange, CA, USA) followed by water rinsing and application of primer and bonding (Optibond FL, Kerr) following the manufacturer’s instructions
**G2: OFL-Ch**	Dentine etching as for G1. Pretreatment with 0.1% Ch water-solution for 1 min and air-drying for 5 s. Application of primer and bonding (Optibond FL, Kerr) following manufacturer’s instructions on the Ch-treated dentine.
**G3: SBU**	Scotchbond Universal (3M ESPE, St. Paul, MN, USA) application in self-etch mode on mineralized dentine according to the manufacturer’s instructions.
**G4: SBU-Ch**	Mineralized dentine treated with 0.1 % Ch for 1 min and air-dried for 5 s. followed by SBU adhesive applied as for Group 3.

**Table 2:** Microtensile bond strengths (mean ± SD, in MPa) of adhesive groups with and without Ch immediately after bonding (T0) and after thermocycling (T1).

	T0	T1
**OFL**	41.3 (14.5)^Aa^ (54.1A/25CC/1.4CD/19.5M)	32.2 (12.9)^Ba^ (66.7A/28CC/1.3CD/4M)
**OFL-Ch**	38.0 (7.7)^Aa^ (61.2A/13CC/2.3CD/23.5M)	29.2 (14.1)^Ba^ (78.3A/6CC/1.2CD/14.5M)
**SBU**	25.0 (16.5)^Bb^ (85.2A/6.2CC/1.2CD/7.4M)	30.4 (11.8)^Aa^ (61.2A/29.6CC/2CD/7.2M)
**SBU-Ch**	28.1 (14.3)^Bb^ (75.5A/9.6CC/2.1CD/12.8M)	33.1 (17.0)^Aa^ (57.7A/27.9CC/1.9CD/12.5M)

For comparison among the adhesive groups with and without Ch for T0 and T1, different lower-case letters in the same columns are significantly different (*p* < 0.05). Different upper-case letters in the same row means a significant difference (*p* < 0.05) in the bond strength between the factor time. Percentages of the failure modes (in parentheses) were classified as: A, adhesive; CC, cohesive in resin composite; CD, cohesive in dentine and M, mixed failure.

**Table 3:** 2 × 4 contingency table comparing nanoleakage distribution between OFL-T0 and OFL-Ch-T0 using the Fisher–Freeman–Halton statistic.

	0%–25%	25%–50%	50%–75%	75%–100%	Total
**OFL-T0**	6	3	1	0	10
**OFL-Ch-T0**	2	0	4	4	10
**Total**	8	3	5	4	20

*P*_A_ = 0.0095, where *P*_A_: the probability of the observed array of cell frequencies plus the sum of the probabilities of all other cell frequency arrays (such as would be consistent with the observed marginal totals) that are equal to or smaller than the probability of the observed array. *P*_B_ = 0.0080, where *P*_B_: the probability of the observed array of cell frequencies plus the sum of the probabilities of all other cell-frequency arrays (such as would be consistent with the observed marginal totals) that are smaller than the probability of the observed array. The difference in the nanoleakage distribution at time T0 between the two groups is significantly different (*p* < 0.05). P_A_ and P_B_ are both non-directional (two-tailed) probabilities. Number of tables evaluated = 112. Note: Chi-square test was not performed on the data set because more than 20% of the cells have an expected frequency of less than 5, and that some cells have an expected frequency smaller than 1.0.

**Table 4:** 2 × 4 contingency table comparing the nanoleakage distribution between OFL-T1 and OFL-Ch-T1 using the Fisher–Freeman–Halton statistic.

	0%–25%	25%–50%	50%–75%	75%–100%	Total
**OFL-T1**	5	4	1	0	10
**OFL-Ch-T1**	1	3	5	1	10
**Total**	6	7	6	1	20

*P*_A_ = 0.0069, where *P*_A_: the probability of the observed array of cell frequencies plus the sum of the probabilities of all other cell frequency arrays (such as would be consistent with the observed marginal totals) that are equal to or smaller than the probability of the observed array. *P*_B_ = 0.0062, where *P*_B_: the probability of the observed array of cell frequencies plus the sum of the probabilities of all other cell frequency arrays (such as would be consistent with the observed marginal totals) that are smaller than the probability of the observed array. The difference in the nanoleakage distribution at time T1 between the two groups is significantly different (*p* < 0.05). P_A_ and P_B_ are both non-directional (two-tailed) probabilities. Number of tables evaluated = 80. Note: Chi-square test was not performed on the data set because more than 20% of the cells have an expected frequency of less than 5, and that some cells have an expected frequency smaller than 1.0.

**Table 5:** 2 × 4 contingency table comparing the nanoleakage distribution between SBF-T0 and SBF-Ch-T0 using the Fisher–Freeman–Halton statistic.

	0%–25%	25%–50%	50%–75%	75%–100%	Total
**SBF-T0**	1	3	1	5	10
**SBF-Ch-T0**	4	1	1	4	10
**Total**	5	4	2	9	20

*P*_A_ = 0.5726, where *P*_A_: the probability of the observed array of cell frequencies plus the sum of the probabilities of all other cell frequency arrays (such as would be consistent with the observed marginal totals) that are equal to or smaller than the probability of the observed array. *P*_B_ = 0.4908, where *P*_B_: the probability of the observed array of cell frequencies plus the sum of the probabilities of all other cell frequency arrays (such as would be consistent with the observed marginal totals) that are smaller than the probability of the observed array. The difference in the nanoleakage distribution at time T0 between the two groups is not significantly different (*p* > 0.05). P_A_ and P_B_ are both non-directional (two-tailed) probabilities. Number of tables evaluated = 88. Note: Chi-square test was not performed on the data set because more than 20% of the cells have an expected frequency of less than 5, and that some cells have an expected frequency smaller than 1.0.

**Table 6:** 2 × 4 contingency table comparing the nanoleakage distribution between SBF-T1 and SBF-Ch-T1 using the Fisher–Freeman–Halton statistic.

	0%–25%	25%–50%	50%–75%	75%–100%	Total
**SBF-T1**	2	5	3	0	10
**SBF-Ch-T1**	3	2	5	0	10
**Total**	5	7	9	0	20

*P*_A_ = 0.5226, where *P*_A_: the probability of the observed array of cell frequencies plus the sum of the probabilities of all other cell frequency arrays (such as would be consistent with the observed marginal totals) that are equal to or smaller than the probability of the observed array. *P*_B_ = 0.4590, where *P*_B_: the probability of the observed array of cell frequencies plus the sum of the probabilities of all other cell frequency arrays (such as would be consistent with the observed marginal totals) that are smaller than the probability of the observed array. The difference in the nanoleakage distribution at time T1 between the two groups is not significantly different (*p* > 0.05). P_A_ and P_B_ are both non-directional (two-tailed) probabilities. Number of tables evaluated = 42. Note: Chi-square test was not performed on the data set because more than 20% of the cells have an expected frequency of less than 5, and that some cells have an expected frequency smaller than 1.0.
